# Comparing the effects of sun exposure and vitamin D supplementation on vitamin D insufficiency, and immune and cardio-metabolic function: the Sun Exposure and Vitamin D Supplementation (SEDS) Study

**DOI:** 10.1186/s12889-015-1461-7

**Published:** 2015-02-10

**Authors:** Mica Hartley, Samuel Hoare, Fiona E Lithander, Rachel E Neale, Prue H Hart, Shelley Gorman, Peter Gies, Jill Sherriff, Ashwin Swaminathan, Lawrence J Beilin, Trevor A Mori, Laura King, Lucinda J Black, Kushani Marshall, Fan Xiang, Candy Wyatt, Kerryn King, Terry Slevin, Nirmala Pandeya, Robyn M Lucas

**Affiliations:** National Centre for Epidemiology and Population Health, The Australian National University, Canberra, Australian Capital Territory 2600 Australia; Department of Health and Human Services, Hobart, Tasmania Australia; University of Canberra, Canberra, Australian Capital Territory Australia; QIMR Berghofer Medical Research Institute, Brisbane, Queensland Australia; Telethon Kids Institute, The University of Western Australia, Perth, Western Australia Australia; Australian Radiation Protection and Nuclear Safety Agency, Melbourne, Victoria Australia; Curtin University, Perth, Western Australia Australia; The Canberra Hospital, Canberra, Australian Capital Territory Australia; School of Medicine and Pharmacology, Royal Perth Hospital Unit, University of Western Australia, Perth, Western Australia Australia; Cancer Council of Western Australia, Perth, Western Australia Australia; University of Queensland, Brisbane, Queensland Australia

**Keywords:** Sun exposure, Vitamin D, Immune function, Cardio-metabolic function, Trial

## Abstract

**Background:**

Adults living in the sunny Australian climate are at high risk of skin cancer, but vitamin D deficiency (defined here as a serum 25-hydroxyvitamin D (25(OH)D) concentration of less than 50 nmol/L) is also common. Vitamin D deficiency may be a risk factor for a range of diseases. However, the optimal strategies to achieve and maintain vitamin D adequacy (sun exposure, vitamin D supplementation or both), and whether sun exposure itself has benefits over and above initiating synthesis of vitamin D, remain unclear.

The Sun Exposure and Vitamin D Supplementation (SEDS) Study aims to compare the effectiveness of sun exposure and vitamin D supplementation for the management of vitamin D insufficiency, and to test whether these management strategies differentially affect markers of immune and cardio-metabolic function.

**Methods/Design:**

The SEDS Study is a multi-centre, randomised controlled trial of two different daily doses of vitamin D supplementation, and placebo, in conjunction with guidance on two different patterns of sun exposure. Participants recruited from across Australia are aged 18–64 years and have a recent vitamin D test result showing a serum 25(OH)D level of 40–60 nmol/L.

**Discussion:**

This paper discusses the rationale behind the study design, and considers the challenges but necessity of data collection within a non-institutionalised adult population, in order to address the study aims. We also discuss the challenges of participant recruitment and retention, ongoing engagement of referring medical practitioners and address issues of compliance and participant retention.

**Trial registration:**

Australia New Zealand Clinical Trials Registry: ACTRN12613000290796 Registered 14 March 2013.

## Background

The incidence of skin cancer is high and rising in most developed countries where the population is predominantly fair-skinned [1]. Indeed, skin cancer is the most common cancer in many of these countries [2,3], a consequence of inappropriately high levels of exposure to ultraviolet (UV) radiation. Sun exposure also has benefits for health such as the initiation of cutaneous vitamin D synthesis. Yet even in locations with high skin cancer incidence, vitamin D deficiency (defined here as a serum 25-hydroxyvitamin D (25(OH)D) concentration of less than 50 nmol/L) is not uncommon [4]. Sun protection programs, developed following recognition of stratospheric ozone depletion and burgeoning skin cancer incidence, are now being challenged to find a balance that minimizes risk of skin cancer but allows sufficient sun exposure to maintain adequate vitamin D status [5]. Recent surveys show that health professionals and the public are confused and concerned about the amount of sun exposure that is optimal for health [6,7]. One option is to encourage sun avoidance and increase vitamin D intake from food or supplements. However, recent studies suggest that there may be beneficial effects of sun exposure through non-vitamin D pathways [8-11]. An alternative strategy is thus to encourage sufficient sun exposure to ensure adequate vitamin D status and provide these potential non-vitamin D-related benefits.

In Australia, current sun exposure guidelines recommend the use of sun protection when the UV Index is 3 or greater. Sun protection includes seeking shade when outdoors, covering up with clothing, using sunscreen on exposed skin, and wearing sunglasses and a hat [12]. Australia has the highest skin cancer incidence in the world: 2 in every 3 Australians will develop a non-melanoma skin cancer by the age of 70 years [3] while the incidence of melanoma of the skin is four-fold higher than the average for more developed regions of the world [13]. Nevertheless, in the most recent Australian Health Survey, 23% of the Australian population aged 12 years and over were vitamin D deficient (25(OH)D concentration of <50 nmol/L), with higher prevalence of deficiency during winter and at higher latitude locations [4]. It is not clear whether current sun protection guidelines are compatible with achieving and maintaining vitamin D adequacy. It is also unclear whether modified sun exposure advice can be safely used to optimise vitamin D status, as an alternative to vitamin D supplementation.

There is considerable indirect evidence that suggests vitamin D has beneficial effects on health, including for several internal cancers, cardiovascular disease, fracture prevention and mental health. Many studies have used latitude, ambient ultraviolet (UV) radiation levels, or history of personal sun exposure as a presumed proxy for vitamin D status (for example, [14]). Other studies have directly measured the serum concentration of 25(OH)D to assess vitamin D status. Many experimental studies show that there are plausible biological pathways, and associations between disease and genes of the vitamin D pathway further implicate vitamin D as important in disease etiology or outcome [15]. However, although low 25(OH)D levels are associated with a range of chronic diseases, vitamin D supplementation trials and meta-analyses of trial data have not generally found a beneficial effect on the health outcomes tested [16].

Sun exposure and, to a lesser degree, latitude and ambient levels of UV radiation, are proxies of vitamin D status. Equally, lower vitamin D status is a marker of a range of other potential disease risk factors, such as low levels of physical activity, or low sun exposure itself. Clinical trials of vitamin D supplementation may be null because the vitamin D doses used are too low, the supplementation duration is for too short a time period, or compliance with the intervention is poor [17]. Alternatively, the results of observational studies may be incorrect because of reverse causation or failure to adequately control for confounding – or because the 25(OH)D level is simply a proxy for sun exposure, time outdoors or associated behaviours. Experimental studies show that sun exposure has positive effects on immune function and cardio-metabolic health, working through both vitamin D and non-vitamin D pathways [8,18,19]. Any positive non-vitamin D pathway effects of sun exposure will not be apparent in vitamin D supplementation trials and may explain the discrepancies between observational studies and clinical trials.

Understanding whether there are separate beneficial effects of sun exposure and vitamin D for health is important for framing public health messages: if non-vitamin D pathways are important, then some sun exposure may be required; if not, then sun avoidance and vitamin D supplementation would achieve vitamin D sufficiency and minimise the risk of UV-induced skin cancer.

The Sun Exposure and Vitamin D Supplementation Study (SEDS Study) was developed to rigorously assess the independent effects of sun exposure and vitamin D supplementation on: i) the management of mild vitamin D deficiency; and ii) relevant markers of immune function and cardio-metabolic health. In this paper we describe the methods of the SEDS Study, including the challenges posed by nationwide participant recruitment, measurement of sun exposure and vitamin D status, provision of vitamin D supplementation and placebo, and maintenance of participant engagement and compliance over a 12-month period.

## Methods/Design

**Figure 1:**
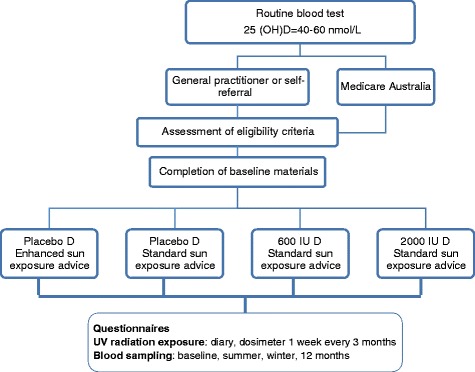
**Study design for the Sun Exposure and Vitamin D Supplementation Study.**

The study design is summarised in Figure 1. Eligible participants are assigned to one of four study regions, according to place of residence: west (Western Australia), east (New South Wales and Australian Capital Territory), north (Queensland), and south (Victoria, Tasmania, South Australia). Within each study region, participants are randomly allocated to one of four study arms (using random number sequences), for a 12-month intervention of:

1. Vitamin D_3_ 2000 IU/day taken orally as a softgel capsule with meals and provision of advice to follow Australian Cancer Council guidelines for sun exposure, which recommend using sun protection if the UV Index is 3 or more. This is the “standard” sun exposure advice (SSEA), and will be a continuation of usual practice for many people.
2. Vitamin D_3_ 600 IU/day taken orally as a softgel capsule with meals and provision of SSEA.
3. Placebo softgel capsule (containing gelatin) and provision of SSEA.
4. Placebo softgel capsule and provision of “enhanced” sun exposure advice (ESEA) guidelines. ESEA guidelines advise the participant to have several brief (10–15 minute) periods of sun exposure each day, protecting the head and neck, but otherwise exposing as much of the body surface as is practicable, without sunscreen. If longer periods of sun exposure are planned, participants are advised to follow Cancer Council Australia guidelines for sun protection. Participants in this arm are provided with a sun exposure monitor that is calibrated for their skin type and delivers an audible warning when the total dose of UV radiation for a day nears one minimum erythemal dose (MED, the dose of UV radiation that results in just perceptible reddening of the skin).

At baseline and three-monthly, participants are asked to complete questionnaires providing information about demographic and personal characteristics, dietary intake, medical history (including medications) and sun exposure over the previous 3 months. At these time points, participants are also asked to maintain a one-week sun exposure, clothing, and physical activity diary and to wear UV dosimeters to measure the received dose of UV radiation. The UV dosimeter is worn at the wrist and is either polysulphone, in which case two dosimeters are supplied, one to be used on a usual working day and the other on a usual non-working day, or an electronic dosimeter to be worn each day for the diary week. Participants have a blood sample taken at baseline, end of study, end of summer and end of winter, with serum and buffy coat (for DNA) aliquots stored at −80°C until analyses are carried out at the completion of the study.

The primary outcome of the SEDS Study is the change in 25(OH)D levels over the 12 months of the trial across the four study arms. Secondary outcomes are changes in blood markers of immune function and cardio-metabolic health across the four arms of the study and in relation to measured sun exposure and 25(OH)D levels.

The SEDS Study is registered with the Australian Clinical Trials Registry (ACTRN 12613000290796) and has been approved by the Human Research Ethics Committees of The Australian National University and The University of Western Australia. Participants are required to provide written informed consent prior to entry into the Study. The SEDS Study has been carefully designed to ensure the safe and ethical conduct of a trial, adequate statistical power, and applicability of the results to the broad range of environmental conditions found across Australia.

### Participant recruitment

Participants are primarily self-referred or recruited through primary care physicians (general practitioners, GPs). In addition, a random selection of adults with a vitamin D test billed to the Australian medical insurance scheme, Medicare, are mailed an invitation to participate in the Study. Once referred, potential participants are assessed for eligibility (see below) against the inclusion and exclusion criteria. If eligible and interested in participating, initial questionnaires and a consent form are mailed out and, on return of the completed material, participants are randomised as described above.

### Eligibility criteria

Eligible participants are aged 18–64 years with a recent 25(OH)D level of 40–60 nmol/L (previous month) and a Fitzpatrick skin type of II-IV [20], determined by questions asked during an initial telephone assessment. Exclusion criteria include current treatment for vitamin D deficiency and medical conditions for which sun exposure and/or vitamin D supplementation may be contraindicated (see below).

## Discussion: considerations informing the study design

In the following sections, the ethical and statistical concerns are discussed with respect to each of the important aspects of the study design.

### Ethical concerns

#### Exclusion of participants with skin type I (eligibility criterion: skin type II to IV).

Fitzpatrick skin types are widely used in photobiology as a measure of sun sensitivity. Skin type is assessed using a brief questionnaire (for example [21]) and categorised as I (very fair) to VI (very dark). There is considerable variability in the minimum erythemal dose (MED, the dose of UV radiation that causes a barely perceptible reddening of the skin) within each skin type category and overlap between the categories [22]. The enhanced sun exposure advice given within the SEDS Study is for a change in the pattern rather than the overall amount of sun exposure. The sun exposure monitor provided to participants in the ESEA arm is set to the individual’s skin type. To allow for the variation in sensitivity to UV radiation within each Fitzpatrick skin type, each monitor is set conservatively to alarm well before the average MED for that skin type. Participants are requested to report any instance where they developed erythema after being outdoors and the sun exposure monitor did not alarm. In this situation, the participant would be instructed to enter one skin type lower into the monitor. Thus, skin type I is excluded from the study, to minimise the risk that participants will be sunburnt while following the ESEA guidelines.

#### Exclusion of participants with a 25(OH)D level less than 40 nmol/L (eligibility criterion: recent vitamin D result of 40–60 nmol/L)

To the best of our current knowledge, there is no harm in delaying treatment of people with a 25(OH)D level of 40–60 nmol/L for one year [23]. If there is a causal association between vitamin D deficiency and disease risks, the increased risk appears to be primarily in those with very low 25(OH)D levels (e.g. <30 nmol/L) [24]. Each participant in the SEDS Study has a one in four chance of being in the placebo vitamin D and SSEA group and will not receive any active treatment. Hence we have chosen a conservative cut-off of 40 nmol/L, below which there is stronger evidence that active treatment for vitamin D deficiency may be required.

#### Exclusion of participants at high risk of skin cancer

Participants with low 25(OH)D levels are likely to have had low recent sun exposure. If they follow the sun exposure advice for their intervention arm, they may receive a higher dose of UV radiation than would have otherwise occurred. Potential participants who report that they are being monitored due to a high risk for skin cancer, have had a previous diagnosis of melanoma or squamous cell carcinoma, or more than five basal cell carcinomas (BCCs) removed in the past five years, are thus excluded from participation. BCC is extremely common in adults in Australia [3] so that it is not practicable to exclude anyone who has had a single previous BCC diagnosed. The ESEA includes advice to always wear a hat and protect the face, neck and ears with sunscreen when outdoors, as most skin cancers arise in these areas [25].

#### Exclusion of medical conditions where sun exposure or vitamin D supplementation may have adverse effects

Although the doses of vitamin D supplementation used in the SEDS Study are relatively low, potential participants with a history of the following medical conditions are excluded from participation, since the intervention could adversely affect their health (or that of their offspring): sarcoidosis, history of renal calculi, uncontrolled endocrine disease, hepatic or renal disease, photosensitivity diseases such as systemic lupus erythematosus with cutaneous manifestations, pregnancy or lactation.

#### Doses of vitamin D supplementation

The United States Institute of Medicine (IOM) Recommended Dietary Allowance of vitamin D for adults aged up to 70 years with limited sun exposure is 600 IU/day [26]. The current recommendation in Australia is 200–400 IU/day (depending on age), with a maximum dose of 3200 IU/day [27]. In the SEDS Study, we have chosen to use the dose recommended by the IOM (i.e., 600 IU/day) as our lower reference point against which to assess the effectiveness of sun exposure in raising 25(OH)D levels, and an upper dose of 2000 IU/day as this has been previously shown to effectively raise 25(OH)D levels without evidence of toxicity over a prolonged period of administration [28].

#### Enhanced sun exposure advice (ESEA) as an intervention

Previous work has shown that the amount of the body surface area that is exposed to the sun strongly influences vitamin D production [29,30]. Prolonged sun exposure can result in degradation of vitamin D in the skin to non-biologically active photo-products [31]. Thus, the ESEA focuses on short, frequent sun exposure, with as much skin exposed as is feasible for the circumstances. If longer periods in the sun are planned, participants are advised to use sun protection if the UV Index is 3 or more. The UV Index is available on daily weather forecasts on television, radio, internet, in most daily newspapers in Australia, and on the Cancer Council Australia website. This information is provided to participants in their sun exposure guidelines.

### Enhancing the statistical power of the SEDS study

The study aims to recruit 228 participants in each group, spread evenly across the four study regions (west, east, north, and south, as described above). Assuming 20% attrition over the one year of the study, this sample size will have 90% power to show equivalence in the post-intervention mean serum levels of 25(OH)D, assuming that equivalence is plus or minus 1/3 of the standard deviation (that is, ± 5 nmol/L) [32].

#### Exclusion of individuals under 18 or over 64 years old (eligibility criterion: aged 18–64 years)

Restricting participation in the SEDS Study to a specific age group limits the generalisability of the findings, and thus requires careful consideration. Elderly people tend to have lower vitamin D status than younger people, possibly due to lower cutaneous stores of the precursor, 7-dehydrocholesterol [33]. In addition, skin cancer incidence increases exponentially with increasing age [3]. We considered that the risks for skin cancer may outweigh the benefits of vitamin D synthesis, for the doses of UV radiation required to achieve and maintain vitamin D sufficiency in older adults. Although there is no clearly defined age cut-off, we excluded people over the age of 64 years from participation in the SEDS Study. Participation in the study is limited to adults due to the greater difficulties involved in relation to consent, recruitment and taking blood from children (<18 years).

#### Exclusion of individuals with 25(OH)D level over 60 nmol/L (eligibility criterion: recent 25(OH)D result of between 40–60 nmol/L)

The change in 25(OH)D level following both exposure to UV radiation and vitamin D supplementation depends on the baseline 25(OH)D concentration [34,35]. Additionally, the increase in 25(OH)D level is not linear, and plateaus at approximately 70–80 nmol/L [36,37]. Participants with a recent 25(OH)D level of >60 nmol/L are excluded from participation, so as to maximise the evidence of the response to sun exposure or vitamin D supplementation.

#### Exclusion of individuals with skin type V or VI (eligibility criterion: skin type II–IV)

Although there is debate about whether more deeply pigmented skin produces less vitamin D following UV irradiation than paler skin, the weight of current evidence suggests that this does occur and is particularly evident for lower doses of UV radiation [34,38]. We have thus chosen to exclude individuals with skin types V and VI from the SEDS Study, although additional work is required to specifically study the needs of people with these darker skin types.

#### Exclusion of individuals currently taking vitamin D supplementation

The doses of vitamin D supplementation chosen are relatively low compared to those used in other supplementation trials. Individuals who are currently taking a daily multivitamin (containing more than 200 IU vitamin D_3_), are asked to switch to one containing no, or low dose, vitamin D in order to be eligible to participate in the Study. The aim is to avoid contamination across the groups, maintaining minimal supplemental vitamin D in the placebo arms and ensuring that the low-dose arm remains as a low-dose, intermediate group.

#### Multicentre trial–participants recruited from across Australia

The SEDS Study is recruiting Australia-wide in order to provide wide variation in levels of ambient solar UV radiation, as well as the amplitude of the seasonal variability, and temperature, which affects patterns of clothing and time outdoors. Australia spans a wide range of latitudinal zones, from Darwin at 12° South to Hobart at 43° South. This results in considerable variation in the typical sun exposure and doses of UV radiation. For example, at the end of June (Southern Hemisphere winter), the maximum UV Index in Darwin in the Northern Territory is around 8, compared to Kingston (15 km from Hobart) in Tasmania, which has a maximum UV Index of around 1. The SEDS Study aims to contribute to the evidence base for messages of optimal sun exposure (amount and pattern) under different climatic conditions, including temperature, humidity and ambient UV radiation. Australia provides a good location to assess this broad range of climatic conditions, within a homogeneous healthcare system and a population that is relatively homogeneous in terms of composition across different regions.

### Challenges in study methodology

#### Recruitment methods

Recruiting nearly 1000 people across Australia with a recently measured 25(OH)D level within a specific range is challenging. Random selection from a population register is not feasible, unless the study then carries out screening for the 25(OH)D level. The main route of recruitment is through GPs who identify potential participants following vitamin D testing undertaken as part of routine clinical care. Research in general practice is known to be difficult due to the time pressures faced in this environment. We have therefore worked hard to initially elicit support and then to maintain engagement using the following methods.

To recruit GPs to assist with the SEDS Study, we use face-to-face meetings with GPs, their practice managers and practice nurses, our personal networks, academic units of general practice attached to universities and medical schools, faxes to regional GP mailing lists, advertisements in GP newsletters, conference flyers, and unannounced visits to general practices. The latter is the least productive, and face-to-face contact the most productive in terms of generating referral of potential participants.

The GPs and their medical practices are provided with information about the study aims, methods and how patients can be referred to the study. In some cases, and only with the permission of the GP, pathology providers have agreed to add a comment to the pathology report for patients with a serum 25(OH)D of 40–60 nmol/L stating that the patient may be eligible for the SEDS Study. GPs receiving the 25(OH)D result seek the patient’s consent to release his/her contact details to the SEDS Study team via fax, email or the SEDS website, including confirmation that the patient has agreed to the release of his/her contact details. To maximise participant recruitment and maintain GP interest and commitment to the Study, researchers visit practices in person throughout all study regions and provide regular study updates via email or post, including relevant publications.

Participants can also self-refer in response to media coverage, the study website (http://www.sedsstudy.org/), or the clinical trials register. However, many of these potential participants are not eligible because they have either not had a recent vitamin D test or are already being treated for vitamin D deficiency. Vitamin D testing is common in Australia, (there were 4 million tests in 2013–4) and has been subsidised by the government medical insurance agency, Medicare. We have used a mail out to a random sample of people who have recently claimed the rebate for a vitamin D test, inviting claimants to contact study personnel to discuss possible participation. We are thus using a multi-pronged approach to maximise recruitment to the SEDS Study.

#### Enhancing accurate data collection

Where possible, the SEDS Study uses validated questionnaires for data collection that can be completed online or in hard-copy. Tools to facilitate the accuracy and standardisation of data collection include online videos and a set of Frequently Asked Questions (FAQs). The online videos describe how to take an accurate measurement of waist circumference, how to use the two types of dosimeter, and also how to complete one of the more complex questionnaires. The FAQs provide guidance for each of the more complicated questionnaires, and are modified as new questions arise.

#### Enhancing compliance

Participant compliance is known to be a challenge in clinical trials [39]. To optimise compliance we contact participants weekly by SMS text message, email or telephone, to remind them to take their study medication and follow their sun exposure advice every day for the entire year. Each week the SMS focuses on one of the elements of the sun exposure guidelines specific to the participant’s intervention group. For the participants in the SSEA groups, these guidelines are the standard “Slip, Slap, Slop, Seek and Slide” messages used by Cancer Council Australia. Participants receiving the ESEA receive specially developed guidelines made to look and sound familiar: Skin (expose as much skin as possible), Short time (brief sun exposures), Slap (wear a hat), Slide (wear sunglasses), Safe (use the sun exposure monitor) and Stride (be physically active). In order to enhance compliance with the sun exposure guidelines, participants are provided with a postcard-sized magnet for the refrigerator that has the specific sun exposure guidelines in a simple, colourful and easy-to-read image so that participants will see the guidelines many times each day.

#### Participant retention over a 12-month period

Each participant is involved in the SEDS Study for one year. In addition to the weekly reminders, study personnel make regular contact by telephone or email. Packages are posted to the participants every three months for data collection, and blood collection forms are sent four times a year; these provide additional opportunities to engage with participants. The SEDS Study website has information about, and photographs of, the study team – since the researchers do not meet the participants, this is an opportunity to make the team as “real” as possible. In addition, a study Facebook page allows participants to communicate with the study team.

Participants who complete the entire 12 months of participation are entered into a draw for one of several shopping vouchers. Since the study will be ongoing over nearly two years, a draw is held for every 200 participants completing the SEDS Study.

### Study outcomes

#### Sun exposure for the management of mild vitamin D insufficiency

Using this intervention study design, the SEDS Study will test whether advice to change the pattern of sun exposure can effectively achieve and maintain vitamin D adequacy (serum 25(OH)D levels of 50 nmol/L or higher) over 12 months. We will also calibrate the effectiveness of ESEA against different doses of vitamin D supplementation. In secondary analyses we will examine actual sun exposure over one year (from questionnaire, diary and dosimeter data) in relation to vitamin D sufficiency, and according to a range of parameters including age, sex, body mass index and levels of physical activity. These findings will have direct clinical applicability for primary care physicians seeing community-dwelling patients with mild vitamin D insufficiency and needing to choose management options.

#### Separate effects of sun exposure and vitamin D on immune and cardio-metabolic function

At the completion of the SEDS Study we will measure, on the stored blood, levels of specific immune and cardio-metabolic markers for which there is past evidence from experimental studies that a vitamin D or a sun exposure effect is likely to be apparent. For immune function, these markers include DNA methylation of the FoxP3 gene as a measure of T regulatory cell number and function [40,41]; total and house dust mite-specific IgE levels [42-44]; varicella zoster IgG (King, unpublished data). Previous work on sun exposure, vitamin D and cardio-metabolic health suggests that the following markers are likely to show an effect of changes in vitamin D or exposure to the sun: fasting lipid profile [45-47]; fasting serum glucose [48-50]; fasting serum leptin and adiponectin [51-53]. The intervention study design has been deliberately chosen to allow us to examine whether ESEA (and higher sun exposure) and vitamin D supplementation (and measured 25(OH)D and vitamin D metabolites) have independent effects on these markers of immune function and cardio-metabolic health.

### What is the potential value of the SEDS Study?

Research in Australia and New Zealand has shown that both health professionals [7,54] and the public [6] are confused about the health benefits and risks of vitamin D supplementation and sun exposure. The SEDS Study has been specifically designed to recruit a population sample to provide an evidence base to clarify some of the issues of concern.

Firstly the study aims to assess and quantify the effect of advice to change the pattern of sun exposure in achieving and maintaining vitamin D adequacy, compared to different doses of vitamin D supplementation. This is an outcome of direct value to clinicians managing vitamin D deficiency.

Secondly, the study builds on recent evidence to suggest that sun exposure and vitamin D may have independent effects on disease risks [10,11,55,56]. In these observational studies, although there is evidence of statistical independence, vitamin D status and sun exposure are closely inter-related, and measurement of both is subject to considerable error. It is difficult therefore to be confident of the existence or relative magnitude of any independent effects. The intervention study design is specifically being used in the SEDS Study to test these factors and to unravel some of the confusion surrounding vitamin D and sun exposure.

## Abbreviations

25(OH)D: 25-hydroxyvitamin D
DNA: Deoxyribonucleic acid
ESEA: Enhanced sun exposure advice
GP: General Practitioner (primary care physician)
IOM: US Institute of Medicine
IU: International Unit
MED: Minimum erythemal dose
nmol/L: Nanomoles per litre
SEDS: Sun Exposure and Vitamin D Supplementation
UV: Ultraviolet
